# Effect of lactoperoxidase on the antimicrobial effectiveness of the thiocyanate hydrogen peroxide combination in a quantitative suspension test

**DOI:** 10.1186/1471-2180-9-134

**Published:** 2009-07-09

**Authors:** A Welk, Ch Meller, R Schubert, Ch Schwahn, A Kramer, H Below

**Affiliations:** 1 Department of Restorative Dentistry, Periodontology and Endodontology, Dental School, University of Greifswald, Greifswald, Germany; 2 Department of Operative Dentistry and Periodontology, Center of Dentistry, Oral Medicine, and Maxillofacial Surgery, University Hospital Tuebingen, Tuebingen, Germany; 3 Private dental practice, Berlin, Germany; 4 Institute of Hygiene and Environmental Medicine, University of Greifswald, Greifswald, Germany

## Abstract

**Background:**

The positive antimicrobial effects of increasing concentrations of thiocyanate (SCN-) and H_2_O_2 _on the human peroxidase defence system are well known. However, little is known about the quantitative efficacy of the human peroxidase thiocyanate H_2_O_2 _system regarding Streptococcus mutans and sanguinis, as well as Candida albicans. The aim of this study was to evaluate the effect of the enzyme lactoperoxidase on the bactericidal and fungicidal effectiveness of a thiocyanate-H_2_O_2 _combination above the physiological saliva level. To evaluate the optimal effectiveness curve, the exposure times were restricted to 1, 3, 5, and 15 min.

**Results:**

The bactericidal and fungicidal effects of lactoperoxidase on Streptococcus mutans and sanguinis and Candida albicans were evaluated by using two test mixtures of a 2.0% (w/v; 0.34 M) thiocyanate and 0.4% (w/v; 0.12 M) hydrogen peroxide solution, one without and one with lactoperoxidase. Following the quantitative suspension tests (EN 1040 and EN 1275), the growth of surviving bacteria and fungi in a nutrient broth was measured. The reduction factor in the suspension test without lactoperoxidase enzyme was < 1 for all three tested organisms. Thus, the mixtures of 2.0% (w/v; 0.34 M) thiocyanate and 0.4% (w/v; 0.12 M) hydrogen peroxide had no in vitro antimicrobial effect on Streptococcus mutans and sanguinis or Candida albicans. However, the suspension test with lactoperoxidase showed a high bactericidal and fungicidal effectiveness in vitro.

**Conclusion:**

The tested thiocyanate and H_2_O_2 _mixtures showed no relevant antimicrobial effect. However, by adding lactoperoxidase enzyme, the mixtures became not only an effective bactericidal (Streptococcus mutans and sanguinis) but also a fungicidal (Candida albicans) agent.

## Background

Maintaining daily oral hygiene is essential to prevent caries, gingivitis, and periodontitis [1-3]. To support mechanical plaque control, which is mostly insufficient [4-6], antiseptics are used in toothpastes and mouth rinses [7-10].

However, the concentrations and frequency of use of antiseptics are limited to avoid side effects, such as discoloration of teeth and tongue, taste alterations, mutations [11,12], and, for microbiostatic active agents, the risk of developing resistance or cross-resistance against antibiotics [13]. Therefore, it would seem better to stimulate or support the innate host defence system, such as the oral peroxidase-thiocyanate-hydrogen peroxide system.

Human saliva contains peroxidase enzymes and lysozyme, among other innate host defence systems. The complete peroxidase system in saliva comprises three components: the peroxidase enzymes (glycoprotein enzyme), salivary peroxidase (SPO) from major salivary glands and myeloperoxidase (MPO) from polymorphonuclear leucocytes filtering into saliva from gingival crevicular fluid; hydrogen peroxide (H_2_O_2_); and an oxidizable substrate such as the pseudohalide thiocyanate (SCN^-^) from physiological sources [14,15]. SPO is almost identical to the milk enzyme lactoperoxidase (LPO) [16,17]. All these peroxidase enzymes catalyze the oxidation of the salivary thiocyanate ion (SCN^-^) by hydrogen peroxide (H_2_O_2_) to OSCN^- ^and the corresponding acid hypothiocyanous acid (HOSCN), O_2_SCN^-^, and possibly O_3_SCN^- ^[18], which have been shown to inhibit bacterial [19-23], fungal [24], and viral viability [25]. However, the system is effective only if its components are sufficiently available in saliva. Salivary concentration of SCN^- ^varies considerably and depends, for instance, on diet and smoking habits. The normal range of salivary SCN^- ^for nonsmokers is from 0.5 to 2 mM (29–116 mg/l), but in smokers [26,27], the level can be as high as 6 mM (348 mg/l). Pruitt et al. [28], for example, see the main limiting component for the production of the oxidation products of SCN^- ^in whole saliva to be the hydrogen peroxide (H_2_O_2_) concentration. Thomas et al. [29] showed that the combination of LPO, SCN^-^, and 0.3 mM (10.2 mg/l) H_2_O_2 _caused complete inhibition that lasted for nearly 16 h, whereas 0.3 mM (10.2 mg/l) H_2_O_2 _alone had no effect. However, if no more H_2_O_2 _was added, the concentration of the inhibitor OSCN^- ^fell because of slow decomposition of OSCN^-^, and, when OSCN^- ^fell below 0.01 mM (0.74 mg/l), the bacteria resumed metabolism and growth. The loss of OSCN^- ^over time is based on decomposition, not on the reaction with bacteria [29].

The typical concentration of peroxidases in whole saliva is roughly 5 μg/ml, whereas the MPO concentration (3.6 μg/ml) is approximately twice the amount of SPO (1.9 μg/ml) [30]. Therefore, even if SPO is deficient, MPO activity would probably be adequate for SCN^- ^oxidation in mixed saliva [30]. The study by Adolphe et al. [31] showed that the lactoperoxidase system's antimicrobial efficiency can be enhanced by better concentration ratios of the LPO system components. However, this finding was postulated for only near physiological conditions and did not consider a concentration of thiocyanate and H_2_O_2 _higher than the physiological one.

Rosin et al. [32] showed that, in the saliva peroxidase system, increasing SCN^-^/H_2_O_2 _above its physiologic saliva level reduced plaque and gingivitis significantly compared to baseline values and a placebo. A new dentifrice formulated on these results showed the same effects regarding plaque and gingivitis prevention in comparison to a benchmark product containing triclosan [33]. However, the effects were not sufficient to recommend using the SPO system to effectively prevent oral diseases in the long run.

Thus, the question arose, Is it possible to increase antimicrobial effectiveness by adding not just thiocyanate and hydrogen peroxide but also LPO to oxidize as much the SCN^- ^anions as possible to become an effective antimicrobial agent? Therefore, we conducted a standardized quantitative suspension test at a fixed concentration level of all three components above the physiological one to evaluate the influence of LPO on the lactoperoxidase-thiocyanate-hydrogen peroxide system relative to its bactericidal and fungicidal effectiveness against Streptococcus mutans and sanguinis and Candida albicans.

## Results

The reduction factors (RF) of the test suspensions without and with LPO on the viability of Streptococcus mutans, Streptococcus sanguinis, and Candida albicans at different time points (1, 3, 5, and 15 min) are shown in tables 1, 2 &3.

**Table 1:** Reduction factors of the test thiocyanate hydrogen peroxide microbial suspension without and with LPO to Streptococcus mutans at different time points.

	Group A				Group B				A vs. B^2^
	Without LPO				With LPO				
Time	Reduction factor	Comparisons within A^1^			Reduction Factor	Comparisons within B^1^			
					
		1 vs. 3	3 vs. 5	5 vs. 15		1 vs. 3	3 vs. 5	5 vs. 15	

[min]	Mean ± SD	p	p	p	Mean ± SD	p	p	p	p

1	0.23 ± 0.26				0.03 ± 0.17				0.128
		0.844				0.016			
3	0.21 ± 0.36				0.53 ± 0.22				0.026
			0.375				0.016		
5	0.25 ± 0.12				7.49 ± 0.64^3^				< 0.001
				0.016				0.375	
15	0.69 ± 0.43				7.41 ± 0.69^3^				< 0.001

1) Wilcoxon test with a significant level of < 0.05

2) Mann-Whitney U test with a significance level of < 0.001

3) Complete killing of all cells in test suspension

**Table 2:** Reduction factors of the test thiocyanate hydrogen peroxide microbial suspension without and with LPO to Streptococcus sanguinis at different time points.

	Group A				Group B				A vs. B^2^
	Without LPO				With LPO				
Time	Reduction factor	Comparisons within A^1^			Reduction Factor	Comparisons within B^1^			
					
		1 vs. 3	3 vs. 5	5 vs. 15		1 vs. 3	3 vs. 5	5 vs. 15	

[min]	Mean ± SD	p	p	p	Mean ± SD	p	p	p	p

1	0.10 ± 0.90				0.13 ± 0.12				0.710
		0.609				0.078			
3	0.16 ± 0.15				0.78 ± 0.67				0.073
			0.109				0.016		
5	0.27 ± 0.17				4.01 ± 3.88				0.073
				0.016				0.063	
15	1.03 ± 0.60				8.12 ± 0.22^3^				< 0.001

1) Wilcoxon test with a significant level of < 0.05

2) Mann-Whitney U test with a significance level of < 0.001

3) Complete killing of all cells in test suspension

**Table 3:** Reduction factors of the test thiocyanate hydrogen peroxide suspension without and with LPO to Candida albicans at different time points.

	Group A				Group B				A vs. B^2^
	Without LPO				With LPO				
Time	Reduction factor	Comparisons within A^1^			Reduction Factor	Comparisons within B^1^			
					
		1 vs. 3	3 vs. 5	5 vs. 15		1 vs. 3	3 vs. 5	5 vs. 15	

[min]	Mean ± SD	p	p	p	Mean ± SD	p	p	p	p

1	0.12 ± 0.19				0.43 ± 0.33				0.077
		0.496				0.004			
3	0.26 ± 0.26				6.78 ± 0.25^3^				< 0.001
			0.141				0.551		
5	0.15 ± 0.13				6.75 ± 0.22^3^				< 0.001
				0.004				1.000	
15	0.93 ± 0.58				6.74 ± 0.26^3^				< 0.001

1) Wilcoxon test with a significant level of < 0.05

2) Mann-Whitney U test with a significance level of < 0.001

3) Complete killing of all cells in test suspension

The accompanying suspension tests with single components (SCN^-^, LPO) and combinations of two components (LPO+SCN^-^, LPO+H_2_O_2_) showed no clinically relevant effects (RF ≤ 0.3) at all time points. Only the single component H_2_O_2 _showed a reduction factor of 1.5 after 15 min.

### Streptococcus mutans

The antibacterial reductions of the thiocyanate-hydrogen peroxide system without LPO increased with time and were statistically significantly different between 5 and 15 min. However, they remained at a very low level (RF < 1). Thus, the suspension without LPO had practically no bactericidal effectiveness. The suspension with LPO showed a distinct antibacterial reduction (RF 7.49) after 5 min, which means the complete killing of all cells. Thus, a further increase of the reduction factor was not possible. The comparison between groups A (without LPO) and B (with LPO) showed a statistically significant difference in favour of group B after 5 and 15 min (Table 1).

### Streptococcus sanguinis

The antibacterial reductions of the thiocyanate-hydrogen peroxide system without LPO increased with time but only to a very low level (RF ≤ 1) with practically no bactericidal effectiveness. The suspension with LPO showed an effective antibacterial reduction after 5 min (RF 4.01 ± 3.88) and after 15 min (RF 8.12 ± 0.22). The RFs between 3 and 5 min were statistically significantly different. The comparison between groups A and B showed a statistically significant difference in favour of B (with LPO) after 15 min (Table 2).

### Candida albicans

The antifungal reduction of the thiocyanate-hydrogen peroxide system without LPO (Group A) increased with time but only to a very low level (RF < 1) with practically no fungicidal effectiveness. The suspension with LPO (Group B) showed an effective fungicidal reduction after 3 min (RF 6.78 ± 0.25), which means the complete killing of all microbes. Thus, a further increase of the reduction factor was not possible.

The RFs between 3 and 5 min were statistically significantly different. The comparison between groups A and B showed a statistically significant difference in favour of B (with LPO) after 3 min (Table 3).

## Discussion

The applied quantitative suspension tests are recognized European norm tests for evaluating bactericidal (EN 1040) and fungicidal efficacy (EN 1275) of a newly developed antiseptic [34,35]. In contrast to common antimicrobial tests (inhibition tests), these quantitative suspension tests facilitate, for example, the strict distinctions between bacteriostatic/fungistatic and bacteriocidal/fungicidal effects by neutralizing the active agent. The tests are also useful for determining a quantitative curve for concentration and time of an antiseptic. Thus, the tests are suitable for evaluating the effect of LPO on the lactoperoxidase-thiocyanate-hydrogen peroxide system's antimicrobial effects. However, the results must be interpreted within the limitations of an in vitro test.

The industrially produced LPO enzyme such as that used in toothpaste [36] was used because of its reproducible quality. Human SPO is slightly different from industrially produced LPO. However, the main characteristics of the industrially produced LPO are identical to saliva peroxidase [16,17]. Based on this similarity, industrially produced LPO is used instead of SPO in studies and is often referred to as LPO in the literature [37].

The efficiency of the LPO system depends – besides the concentration of its components – on exposure time and pH value [29,31]. Therefore, to determine when the LPO system or the oxidation products reached their initial optimal bactericidal and fungicidal effectiveness, tests were conducted at the exposure times of 1, 3, 5, and 15 min.

All tests were conducted at the pK_a _(pH 5.3) of HOSCN/OSCN^- ^[38], because pretests showed that the lactoperoxidase-thiocyanate-hydrogen peroxide system was effective at 5.3 pH. Lumikari et al. [23] found the optimum pH to be about 5.0. Increasing the HOSCN/OSCN^- ^concentration by adding H_2_O_2 _could raise the inhibition of Streptococcus mutans in human saliva [21,36] but only at a pH around 5 and not at neutral pH because of the shift of OSCN^- ^to HOSCN by a low pH value in favour of HOSCN. Unlike OSCN^-^, HOSCN has no charge, which facilitates penetration through the lipophilic bacterial cell membrane and raises the antimicrobial effectiveness of the saliva antiperoxidase system [18]. Thus, the most effective product of the LPO system works around the pH, where the biofilm/saliva pH level is pathologically effective.

To completely ensure that the tested effect of the lactoperoxidase enzyme on the thiocyanate-hydrogen peroxide system above the physiological concentration level was not based primarily on single components (H_2_O_2_, SCN^-^, LPO) or on combination of two components (LPO+SCN^-, ^LPO+H_2_O_2_), accompanying suspension tests were conducted.

With one exception, all accompanying single component tests showed no clinically relevant antimicrobacterial effectiveness (RF: ≤ 0.3). Only the single component H_2_O_2 _showed a moderate reduction factor of 1.5 after 15 min. This result is in line with the known bactericidal effect of H_2_O_2 _[29]. However, in combination with LPO, the effect of H_2_O_2 _was reduced compared to its single effect. We assume that the radicals, which are produced by the reaction of LPO with H_2_O_2 _[39], are short-lived intermediates that cannot react bactericidally under the test conditions.

All suspension tests without LPO at all time points showed no or no clinically relevant antimicrobial effectiveness (highest RF: Streptococcus mutans 0.6, Streptococcus sanguinis 1.0, and Candida albicans 0.9). The low reduction potential could be based on H_2_O_2 _itself or, to a small extent, on the oxidation without enzyme of SCN^- ^to OSCN^- ^by H_2_O_2_, especially at higher exposure times.

On the other hand, all suspensions with LPO showed remarkably high antimicrobial effectiveness. In the quantitative suspension test, the lactoperoxidase-thiocyanate-hydrogen peroxide system (group B) showed its maximal reduction (complete) of Streptococcus mutans (RF 7.49) after a 5-min incubation time. Both reduction factors (after 5 and 15 min) were statistically significantly different from group A (without LPO).

The results show the large effect of the LPO enzyme on antibacterial effectiveness of the lactoperoxidase-thiocyanate-hydrogen peroxide system, which can be a powerful bactericide, not just bacteriostatic, if all components are above their physiological levels. It is assumed that the effect is based on not just the described shift of OSCN^- ^to HOSCN (pH 5.3) [38] but also a higher amount of the more effective LPO-caused oxidation products, O_2_SCN^- ^and O_3_SCN^- ^[21,23,28].

In the case of Streptococcus sanguinis, the reduction factor at 5 min (RF 4.01) was statistically significantly higher in comparison with the reduction factor at 3 min (RF 0.78) of Group B (with LPO). However, there was no statistically significant difference between the reduction factors at 5 min in either group (A and B), despite a great difference in their mean values. The reason was the large standard deviation of in RF (4.01 ± 3.88).

We assume that, when the 5-min measurement was taken, the bactericidal effect by HOSCN/OSCN^- ^was already occurring in some experiments but not yet in others. One of the reasons could be the NAD(P)H-OSCN^- ^oxidoreductase system, which Streptococcus mutans and Streptococcus sanguinis and other bacteria have. This system can reduce HOSCN/OSCN^- ^to the less effective components, SCN^- ^and H_2_O_2_. Streptococcus sanguinis has more of this reducing enzyme than does Streptococcus mutans. Thus, we assume that a higher concentration of HOSCN/OSCN^- ^is needed to achieve a similar bactericidal effect on Streptococcus sanguinis than on Streptococcus mutans [40,41], meaning more time in the experiment. After 15 min, the test suspension with LPO had a similar antibacterial effectiveness on Streptococcus sanguinis (RF 8.12 ± 0.22) as on Streptococcus mutans (RF 7.41 ± 0.69).

Rosin et al. [32] used more than the physiological level of SCN^-^-H_2_O_2 _in a toothpaste to increase the human oral defence system. This toothpaste reduced gingivitis and inhibited plaque. The enhancement of these effects by an optimal combination not only of H_2_O_2 _and thiocyanate, but also of LPO enzyme, for mouth rinses or toothpaste formula is certainly possible and should be considered in further clinical studies.

In our study, the LPO system was bactericidal at pH 5.3 to Streptococcus mutans and sanguinis. However, experiments by Thomas et al. [29] showed that the LPO system was effectively bacteriostatic, but not bactericidal, at pH 7 during a 1-h incubation. This finding may mean that the LPO system might shift from bacteriostatic to bactericidal at a point when the Streptococcus mutans causes low pH (<5.5), leading, for example, to demineralisation of tooth hard substances. Thus, the system could be a reservoir, getting its highest antibacterial activity when it is most needed: at a point when pH falls as a result of bacterial lactic acid production.

After 3 min, the reduction of Candida albicans in the test suspension with LPO was already complete. Thus, of the three tested microorganisms, Candida albicans was most sensitive to the lactoperoxidase-thiocyanate-hydrogen peroxide system, even if it was buffered by phosphate. Majerus and Courtois [42], as well as Samant et al. [43], could not find a sufficient antifungal effect of the SCN^-^-H_2_O_2_-LPO system. Lenander-Lumikari [22] found that C. albicans is sensitive to HOSCN/OSCN^-^, but saliva and salivary concentrations of phosphate blocked the antifungal effect of the peroxidase systems. However, they used all components of this system at the physiological human saliva level.

Thus, the lactoperoxidase-thiocyanate-hydrogen peroxide system can be not only fungistatic [44] but also fungicidal for Candida albicans; independently, it is phosphate-buffered at salivary concentrations or higher.

C. albicans can be isolated from the mouth of most individuals, but the fungus causes oral disease such as oral mucositis in primarily immunocompromised individuals [45-47]. Further, Candida albicans is seen as a reservoir for pneumonia [48] and intestinal related diseases [49].

Theraud et al. [50] showed that chlorhexidine was fungicidal on pure cultures, yeast mixtures, and biofilms above a concentration level of 0.5% (w/w). However, Pitten et al. [51] showed that treatment with a 0.3% (w/w) chlorhexidine-based product did not provide a clinical benefit for cancer patients with chemotherapy-induced leukopenia. In their study, the risk of mucositis and clinical sequelae (e.g., C-reactive protein) seemed to be enhanced by chlorhexidine mouth rinse, although the counts of microorganisms on the oral mucous membranes were significantly reduced. They assumed that the reason was the reduced tissue tolerance to chlorhexidine. This assumption is supported by a study that showed a discrepancy between antiseptic activity and clinical effect on radiation-induced [52] or chemo-induced mucositis [53] by chlorhexidine mouth rinse compared with placebo. In a peritoneal explant test for evaluating tissue tolerance, chlorhexidine showed the highest cytotoxicity in comparison to an essential oil and an amine/stannous fluoride mouth rinse [54]. Thus, it could be interesting to increase host innate defence systems, such as the lactoperoxidase-thiocyanate-hydrogen peroxide system, which have no or low effectiveness at the physiological level, by increasing their level of concentration instead of using common antiseptics.

## Conclusion

In summary, in the quantitative suspension test, the SCN^- ^and H_2_O_2 _mixture above normal physiological saliva levels showed little or no antimicrobial effect within 15 min. However, by adding lactoperoxidase enzyme, the tested mixtures became not only an effective bactericidal (Streptococcus mutans and sanguinis) but also a fungicidal (Candida albicans) agent. Thus, all three components of the LPO-system are needed for its microbicidal effect. Subsequent studies should consider loading tests with human saliva and different concentrations of all three components.

## Methods

The study was performed based on the European norms (EN) 1040 and EN 1275. A 9.9-ml test solution (with and without LPO) was mixed with a 0.1-ml bacteria or fungus suspension (overnight culture) and stored at 37°C.

After 1, 3, 5, and 15 min contact time, the test mixture was again well mixed (vortexed), and 1 ml was transferred into 9 ml of neutralizer (polysorbate 80 30 g/L, lecithin 3 g/L, L-histidine 1 g/L, sodium thiosulfate 5 g/L, aqua bidestillata ad 1000 mL). The neutralizer was tested in a prestudy according to the recommended neutralization test of the German Society for Hygiene and Microbiology (DGHM).

After 5 min of neutralization time, 1.0 ml of the neutralized test suspension was mixed with 9.0 ml of dilution solution, and 0.1 ml of this final solution was spread on tryptone soya agar (TSA, Oxoid, Germany) plates. After 42–48 h of aerobic incubation at 36°C (± 1°C), macroscopically visible colonies were counted on the plates. The arithmetic means of the duplicates were calculated with the plates of 15–300 colony-forming units (cfu) as recommended by European norms. Every trial was conducted separately seven times, and the arithmetic means with the corresponding standard deviations were calculated.

Before each experiment was conducted, all components were prepared as follows.

### Test organisms

Preservation and culture of the test organisms (Streptococcus mutans ATCC 35668, sanguinis ATCC 10556, and Candida albicans ATCC 10231) were conducted corresponding largely to EN 1040 and EN 1275 (adjusted number of cells in the suspension: 1.5 × 10^8 ^– 5.0 × 10^8 ^cfu/ml for bacteria and 1.5 × 10^7 ^– 5.0 × 10^7^cfu/ml for fungi).

### Solutions of test mixtures

Buffer adjusted to pH 5.3: 7 parts 0.2 M KH_2_PO_4_, 1 part 0.2 M K_2_HPO_4_; SCN^- ^solution (2% w/v; 0.34 M): 2.8 g NaSCN/100 ml freshly glass-distilled water; H_2_O_2 _solution (0.4% w/v; 0.12 M): 1.12 g carbamide peroxide (CH_4_N_2_O.H_2_O_2_)/100 ml glass-distilled water (prepared immediately before the trial); buffer-LPO solution: 5.0 mg LPO (210 U/mg, Fluka) dissolved in 0.250 ml glycerine and 0.250 ml phosphate buffer saline solution, adding 5 ml of the buffer to pH 5.3.

### Test mixtures and control

Group A contained 5.0 ml buffer solution (pH 5.3), 2.5 ml SCN^- ^solution (2.0% w/v; 0.34 M), and 2.5 ml H_2_O_2 _solution (0.4% w/v; 0.12 M); Group B contained 4.0 ml buffer solution (pH 5.3), 2.5 ml SCN^- ^solution (2.0% w/v; 0.34 M), 2.5 ml H_2_O_2 _solution (0.4% w/v; 0.12 M), and 1 ml buffered-LPO solution. Thus, the LPO concentration in this solution was 83 mg/ml.

The control group contained 5.0 ml buffer solution (pH 5.3) and 5.0 ml water with standardized hardness. All prepared solutions were stored at 37°C until use.

In the same manner, all single components (H_2_O_2_, SCN^-^, LPO) or their combinations (LPO+SCN^-^, LPO+H_2_O_2_) were tested for their antimicrobial effects in accompanying suspension tests.

### Statistical analysis

The microbial counts were expressed as their decimal logarithms. The reduction factor (RF) was calculated as follows:

where cfu c = number of cfu per ml control medium (water with standardized hardness), and cfu t_A/B _= number of cfu per ml test group A or B.

The comparisons at the time points between groups A and B (without and with LPO, respectively) were performed with the Mann-Whitney U test and within groups with the Wilcoxon test. All statistical analyses were carried out with SPSS 11.5.

## Authors' contributions

AW, HB, and AK participated in the design and coordination of the study, supervised the study, and analyzed the data. RS performed most of the laboratory work with the assistance of ChM and HB. ChS carried out the statistical analysis. AW wrote the manuscript. All authors read and approved the final version of the manuscript.
